# Human Y chromosome haplogroup L1-M22 traces Neolithic expansion in West Asia and supports the Elamite and Dravidian connection

**DOI:** 10.1016/j.isci.2024.110016

**Published:** 2024-05-17

**Authors:** Ajai Kumar Pathak, Hovann Simonian, Ibrahim Abdel Aziz Ibrahim, Peter Hrechdakian, Doron M. Behar, Qasim Ayub, Pakhrudin Arsanov, Ene Metspalu, Levon Yepiskoposyan, Siiri Rootsi, Phillip Endicott, Richard Villems, Hovhannes Sahakyan

**Affiliations:** 1 Estonian Biocentre, Institute of Genomics, University of Tartu, 51010 Tartu, Estonia; 2 Department of Human Genetics, KU Leuven, 3000 Leuven, Belgium; 3 Armenian DNA Project at Family Tree DNA, Houston, TX 77008, USA; 4 Department of Pharmacology and Toxicology, Faculty of Medicine, Umm Al-Qura University, Makkah 21955, Saudi Arabia; 5 Monash University Malaysia Genomics Platform, School of Science, Monash University, Bandar Sunway, Selangor Darul Ehsan 47500, Malaysia; 6 Chechen-Noahcho DNA Project at Family Tree DNA, Kostanay 110008, Kazakhstan; 7 Laboratory of Evolutionary Genomics, Institute of Molecular Biology of National Academy of Sciences of the Republic of Armenia, Yerevan 0014, Armenia; 8 Department of Archaeology and Anthropology, Bournemouth University, Fern Barrow, Poole, Dorset BH12 5BB, UK; 9 Department of Linguistics, University of Hawai’i at Mānoa, Honolulu, Hawai’i 96822, USA; 10 DFG Center for Advanced Studies, University of Tübingen, 72074 Tübingen, Germany

**Keywords:** genomic analysis, sequence homology, genomics, paleogenetics, human geography

## Abstract

West and South Asian populations profoundly influenced Eurasian genetic and cultural diversity. We investigate the genetic history of the Y chromosome haplogroup L1-M22, which, while prevalent in these regions, lacks in-depth study. Robust Bayesian analyses of 165 high-coverage Y chromosomes favor a West Asian origin for L1-M22 ∼20.6 thousand years ago (kya). Moreover, this haplogroup parallels the genome-wide genetic ancestry of hunter-gatherers from the Iranian Plateau and the Caucasus. We characterized two L1-M22 harboring population groups during the Early Holocene. One expanded with the West Asian Neolithic transition. The other moved to South Asia ∼8-6 kya but showed no expansion. This group likely participated in the spread of Dravidian languages. These South Asian L1-M22 lineages expanded ∼4-3 kya, coinciding with the Steppe ancestry introduction. Our findings advance the current understanding of Eurasian historical dynamics, emphasizing L1-M22’s West Asian origin, associated population movements, and possible linguistic impacts.

## Highlights

• The Y chromosome haplogroup L1-M22 originated in West Asia around 20,600 years ago
• A group of L1-M22 harboring population expanded with West Asian Neolithic transition
• Another one moved to South Asia, likely participating in Dravidian languages’ spread
• Their descendants expanded in South Asia around 4,000 to 3,000 years ago

## Graphical abstract

**Figure:**
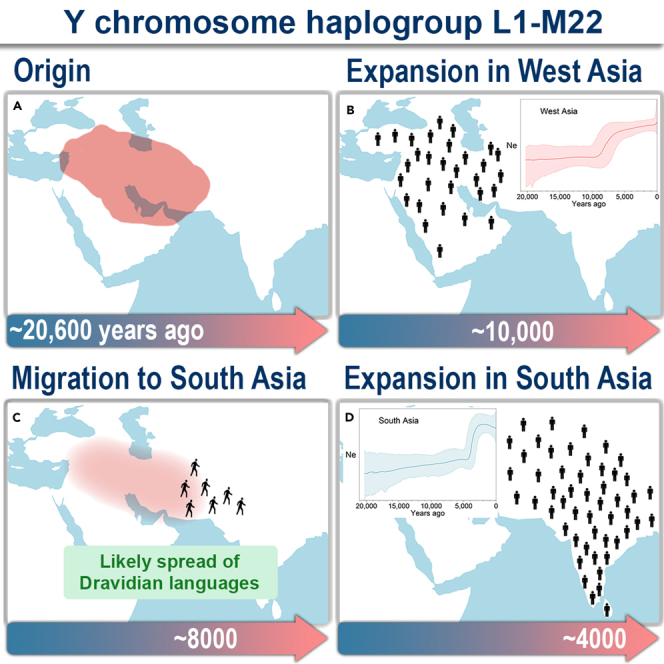

## Introduction

Today’s world bears profound imprints of past human communities in West and South Asia. After the major out-of-Africa migration, modern humans first appeared in West Asia,^1^ then shortly after, in South Asia.^2^ The Y chromosome haplogroups J-M304 and G-M201 inform us about the genetic legacy of these early migrations in West Asia, while the H-M69 in South Asia.^3^^,^^4^^,^^5^ Similarly, mitochondrial DNA haplogroups U, J, and T echo the early exodus in West Asia, while many haplogroups within the macro-haplogroups M, N, and R represent South Asia.^6^^,^^7^^,^^8^^,^^9^^,^^10^ The genome-wide variation also shows distinct spatiotemporal patterns in these regions.^11^^,^^12^^,^^13^ After these early events, the regions underwent the Last Glacial Maximum (LGM), a challenging period around 26.5 to 19 kya.^14^ The LGM appears to have had a more pronounced impact on West Asian human populations.^7^^,^^8^^,^^15^^,^^16^ Unfortunately, no ancient DNA (aDNA) study has been conducted with samples originating from such a deep time of the regions.

The Neolithic demographic transition, a crucial shift in human history,^17^ involved the domestication of plants and animals, leading to increased sedentism, population growth, and the development of complex social structures.^18^^,^^19^ This transition started in the Fertile Crescent in West Asia at ∼12 kya, representing the earliest known instance globally.^20^^,^^21^^,^^22^ By this time, hunter-gatherer populations from present-day Iran and the Caucasus, Anatolia, and the southern Levant accumulated substantial genetic differences and adopted the new lifestyle probably independently.^23^^,^^24^^,^^25^^,^^26^^,^^27^^,^^28^^,^^29^^,^^30^^,^^31^ Populations from present-day Iran and the Caucasus started mixing with Anatolian populations earlier during the Neolithic period, while gene flows with Levantine populations occurred later.^27^^,^^28^^,^^31^ Agricultural practices were gradually reaching other regions. In Europe and Central Asia, the transmission was driven by population expansions from West Asia.^30^^,^^32^^,^^33^^,^^34^^,^^35^ In South Asia, the nature of the Neolithic transition remains elusive. Uncertainties persist regarding whether it transpired through population expansion from neighboring regions^27^^,^^36^^,^^37^^,^^38^^,^^39^^,^^40^^,^^41^ or proceeded through cultural adoption or local developments without substantial population movement.^42^^,^^43^^,^^44^^,^^45^^,^^46^ The Neolithic started later in South Asia than in West Asia. The earliest Neolithic site in South Asia, Mehrgarh, dating to ∼9 kya, exhibits similarities to West Asian Neolithic cultures and is located near West Asia, specifically in Balochistan, present-day southwestern Pakistan.^44^^,^^45^^,^^47^ This region later hosted the beginnings of the sophisticated Indus Valley civilization (IVC). In other South Asian regions, the Neolithic transition unfolded even later, yet featuring distinctive developments indicative of alternative pathways of independent evolution.^44^^,^^45^^,^^47^ It is noteworthy, however, that despite the importance of understanding the South Asian Neolithic, there is a notable lack of aDNA studies. Reconstructions with younger ones find no shared ancestry with Anatolian Neolithic (AN) farmers but rather the presence of ancestry shared with Caucasus/Iranian hunter-gatherers (CIHG).^30^^,^^48^ The South Asian Neolithic is primarily studied in conjunction with the Chalcolithic and Bronze Age periods and in the context of language spread, a topic explored further in the next paragraph.

In the Chalcolithic and Bronze Ages, West and South Asia underwent large-scale population movements,^27^^,^^30^^,^^49^^,^^50^^,^^51^ coinciding with the time when the large language families were spread. In West Eurasia, these were Afro-Asiatic^52^ and Indo-European,^27^^,^^30^^,^^50^^,^^51^^,^^53^^,^^54^ among others. Currently, South Asians predominantly speak Indo-European or Dravidian languages and adhere to the hierarchical caste system and Hinduism.^55^^,^^56^^,^^57^ Indo-European languages, prevalently spoken in northern regions, likely arrived from West/Central Asia during the Middle-Late Bronze Age and have been associated with the origin of the caste system.^30^^,^^41^^,^^50^^,^^58^ On the contrary, Dravidian languages are predominant in southern India and Sri Lanka. Some isolated groups of speakers also live in southwestern Pakistan (Brahui) and northern India. The origin of Dravidian languages remains highly debated. They have been argued to have either an indigenous origin^46^^,^^59^ or linked to the Neolithic dispersals from West Asia, as summarized in the Elamo-Dravidian hypothesis.^36^^,^^37^^,^^38^^,^^40^^,^^41^^,^^42^^,^^60^ The hypothesis postulates linguistic and cultural connections between the extinct Elamite language spoken in ancient Elam (present-day southwestern Iran) and Dravidian languages. Frequently, the Indus Valley or Harappan civilization is suggested as an essential step for the spread of Dravidian languages, meaning that the language of this civilization was a related one. Certain linguistic studies have scrutinized the proposed connection, acknowledging Elamite as a language isolate.^61^ A recent aDNA study suggests that the Iranian ancestral component in the IVC people came from individuals related to, but distinct from, Iranian farmers.^30^ The contributing group lacked AN-related ancestry, common in Iranian farmers after ∼8 kya. These CIHG-related individuals may have arrived in the Indus Valley before the advent of farming there and at ∼7.4–5.7 kya, before the mature IVC mixed with people related to Indian hunter-gatherers (AASI), making a population called the “Indus Periphery Cline” (IPC). A population from IPC later mixed once more with AASI, giving birth to the ancient South Indian (ASI) ancestry,^62^ which is currently widespread in southern Indian populations. However, a question remains: Which population of the two (CIHG-related or AASI) initially spoke a Dravidian language? Another aDNA study directly analyzed a Harappan genome, pushing the split between Iranian farmers and the CIHG-related group back to ∼12 kya.^48^ Unfortunately, these conclusions are based on a single individual, and direct dating was impossible. Moreover, the study was criticized for improperly modeling population history.^63^

**Figure 1:**
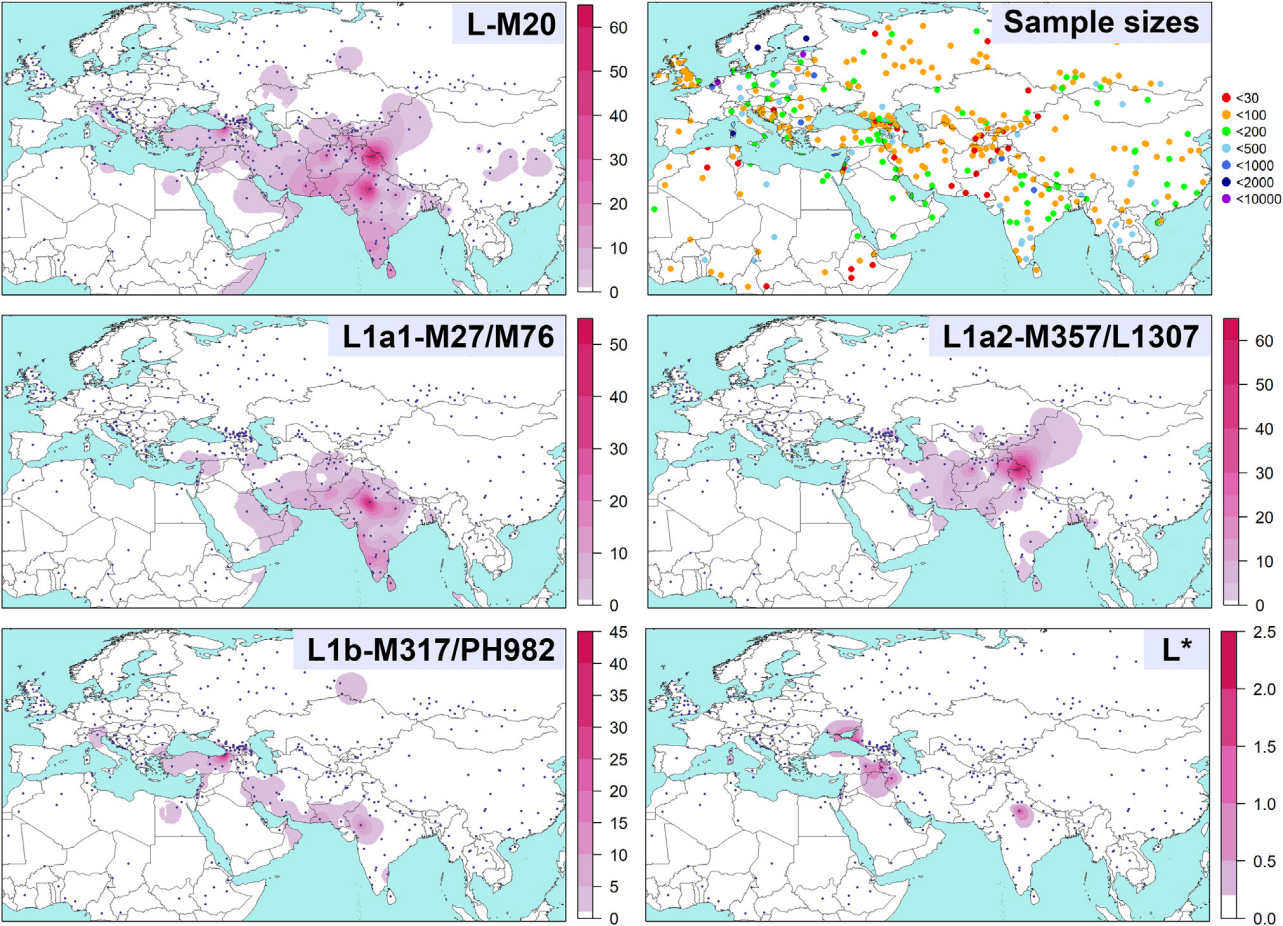
Spatial distribution maps of the haplogroup L-M20 and its branches The frequency maps presented here are generated using data from Table S1. The datasets used for the frequency plots in these maps may differ. Blue-filled circles indicate sampling locations. Scale bars represent frequencies in percentages. Please be aware of the difference in scales between the maps. The figures were created using RStudio software.^66^^,^^67^ The contour map was obtained from http://tapiquen-sig.jimdo.com.

The human Y chromosome haplogroup L-M20 holds significant potential as an avenue for unraveling the complex dynamics of ancient population interactions. M20, M11, M61, and other bi-allelic markers define this haplogroup.^64^^,^^65^ It occurs more in South Asia but also in West Asia, Central Asia, and Europe (Figure 1). The haplogroup splits from its sister branch T ∼45 kya.^3^^,^^4^^,^^5^ Commercial Y chromosome whole sequencing efforts, as documented by phylogenetic trees of the Y Full (YFull) (v12.00.00, https://www.yfull.com/tree/L) and Family Tree DNA Discover (FTDNA) (accessed on 20-03-2024, https://discover.familytreedna.com/y-dna/L-M20/tree), found that at ∼23.5 kya, the haplogroup L-M20 diverged into haplogroups L1 and L2 defined by M22^64^ and L595 markers, respectively. The L1-M22 is the major branch so far, while L2-L595 is rare. Whole high-coverage Y chromosome studies included a small number of L1-M22 samples.^3^^,^^4^^,^^5^^,^^68^ They have estimated the time to the most recent common ancestor (TMRCA) of L1-M22 to be around the LGM. Nevertheless, limited attention has been given to this haplogroup’s origin, population dynamics, and migration patterns. Genotyping studies found three main branches defined by M317,^46^ M27 or M76,^65^ and M357^46^ bi-allelic markers. All these branches fall within the L1-M22. A small number of individuals fall within paragroup L∗. Studies conclude that the haplogroup L-M20 may represent early modern human populations in South Asia.^43^^,^^69^^,^^70^^,^^71^ Others suggest its later migration to South Asia from West Asia with the Neolithic demographic expansion.^72^^,^^73^ The earliest ancient individuals affiliated with this haplogroup substantially postdate its age. They are found in the late-Neolithic/Chalcolithic (∼6.6 kya) in present-day Turkmenistan in a site bordering present-day Iran and in the Chalcolithic (∼6.1 kya) in present-day Armenia within the Caucasus region.^27^^,^^74^ Other individuals living before the common era (BCE) are found in the North Caucasus, present-day Iran, Greece, Turkey, Uzbekistan, Israel, and Pakistan.

In this study, we applied the Bayesian approach to unravel the unfolded dimensions of the haplogroup L1-M22 within South and West Asia. Specifically, we analyzed 165 high-coverage whole Y chromosome sequences to reconstruct the haplogroup’s detailed phylogeny and demographic history. We conducted a statistically robust Bayesian phylogeographic analysis to infer the region where the haplogroup L1-M22 and its various lineages might have originated. Furthermore, we placed published ancient haplogroup L1-M22 genomes in the reconstructed phylogeny. Lastly, we compared our results to the published genetic, archaeological, and linguistic studies relevant to the conclusions about the origin and spread of the haplogroup L1-M22 and its branches.

## Results

Human Y chromosome haplogroup L-M20 and its major branch L1-M22 exhibit a predominant distribution in South and West Asia (Figure 1 and Table S1). In Central Asia, its prevalence is higher in the southern slopes adjacent to South Asia and Iran. This haplogroup remains rare in Europe, primarily concentrated in the central and eastern Mediterranean regions. Although this haplogroup’s branches occur in both South and West Asia, the L1a1-M27/M76 and L1a2-M357/L1307 tend to have a higher frequency in South Asia. In contrast, the L1b-M317/PH982 branch is more prevalent in West Asia.

By using 165 high-coverage whole Y chromosome sequences, we reconstructed the phylogeny of the haplogroup L1-M22. To improve our reconstruction, we included eleven sequences from other haplogroups. We called 4384 high-quality SNPs in the L1-M22 section of the phylogeny. Out of these SNPs, we observed that 0.4% (17) define more than one branch. The proportion of recurring SNPs aligns with the findings of other studies.^3^^,^^16^^,^^75^

**Figure 2:**
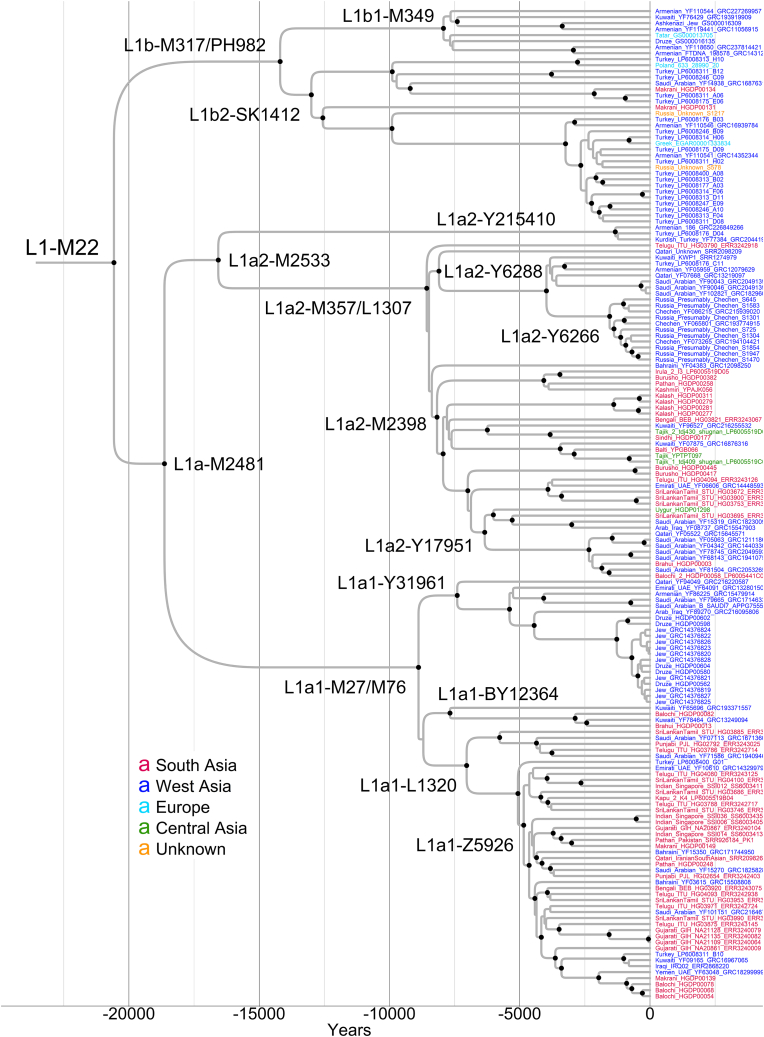
Bayesian phylogenetic tree of the haplogroup L1-M22 Black-filled circles mark the nodes with posterior probability ≥0.95. Sample IDs indicate population affiliation if the original publication explicitly states this; otherwise, the collection country is provided. See also Figure S1, Tables S2 and S4, and File S1.

Utilizing a published calibration point,^3^ we estimated the TMRCA of haplogroup L1-M22 to be ∼20.6 kya, with 95% HPD interval of 17.9–23.1 kya (Figure 2 and File S1). Our estimate aligns with those suggested previously^5^^,^^68^ and overlaps with the Last Glacial Maximum (LGM) (26.5–19.0 kya).^14^ The resulting Y chromosome mutation rate corresponds to 6.78e^−10^ mutations bp^−1^ year^−1^ (95% HPD = 6.04e^−10^–7.57e^−10^), which is comparable with earlier estimates.^3^^,^^16^^,^^76^

Despite our best efforts, we were unable to differentiate the SNPs that define the L1-M22 branch from those defining the L-M20 branch because we needed more data from other L-M20 branches. While most haplogroup L-M20 members known to date belong to the L1-M22 branch, some sources indicate the existence of at least one L-M20(xM22) lineage. The YFull includes three present-day individuals in the L2-L595 lineage, with residences in Lebanon, Turkey, and the Italian island of Sardinia. Additionally, the FTDNA features more individuals from Sardinia and one from the USA. aDNA studies have revealed additional individuals in this lineage, most of whom trace their origins to West Asia. All individuals exhibit CIHG ancestry; many also have AN and/or Levantine Neolithic (LN) ancestry. One individual of non-West Asian origin is found in Europe. Genotyping studies have also reported individuals that belong to the haplogroup L-M20 but do not fall in branches defined by M27/M76, M357, or M317 markers (Figure 1 and Table S1). This suggests a potential association with the L2-L595 lineage or even an unknown L-M20(xM22) lineage. Alternatively, they could belong to the rare L1a2-Y215410 sub-branch within the L1-M22 (Figures 2 and S1).

Our phylogenetic analysis unveils a detailed framework, as Figures 2 and S1 illustrate. Initially, L1-M22 bifurcates into two primary branches, L1a-M2481 and L1b-M317/PH982. The L1a-M2481 further diverges into L1a1-M27/M76 and L1a2-M2533, which, in turn, splits into L1a2-Y215410 and L1a2-M357/L1307. We will comprehensively explore these major branches in the subsequent paragraphs.

The L1a1-M27/M76 splits starting at ∼8.9 kya, giving rise to three sub-branches (Figures 2 and S1). The L1a1-Y31961 is strictly West Asian and coalesces at ∼7.4 kya, including individuals from the Armenian Highland, the Levant, and the Arabian Peninsula. The L1a1-L1320 sub-branch is widespread in southern (more) and northern (less) India, Pakistan, and Bangladesh, with a small number of samples also present in West Asia. The L1a1-BY12364 sub-branch is minor, represented by only two individuals from Kuwait and two from Pakistan. Genotyping analyses confirm that L1a1-M27/M76 occurs widely in South and West Asia. As mentioned above, South Asian populations exhibit higher frequencies than West Asian ones (Figure 1 and Table S1).

The L1a2-M357/L1307 splits starting at ∼8.6 kya, similar to the L1a1-M27/M76 (Figures 2 and S1). It yields two singleton lineages and two sub-branches. The singleton lineages originate from West and South Asia. The L1a2-Y6288 sub-branch is strictly West Asian and coalesces at ∼8.1 kya. It includes individuals from the Arabian Peninsula, the Armenian Highland, and Anatolia. It also contains a recently diversified lineage (L1a2-Y6266, ∼1.6 kya) with members from the Russian Federation. We need population information on many of these individuals, but we know three are Chechens from the Northeast Caucasus. Genotyping studies have shown that L1a2-M357/L1307 is found mainly among Chechens and Ingushes from the Northeast Caucasian populations and is rather frequent there.^77^^,^^78^ Furthermore, in the YFull, the L1a2-Y6266 lineage predominantly comprises individuals from the Chechen and Ingush populations. It corresponds to the L-Y6248 in the FTDNA and includes individuals from the same populations. Therefore, the L1a2-Y6266 lineage is specific to these Nakh-Dagestanian-speaking populations. Importantly, this lineage coalesces in the West Asian part of the L1a2-M357/L1307, contradicting its migration from South Asia.^78^ Unfortunately, it is hard to say anything conclusive about the more specific origin of this lineage as it splits at ∼4 kya from other lineages. The L1a2-M2398 sub-branch coalesces at ∼8.2 kya and occurs mainly in South Asia. It includes many individuals from southern and northern India, Pakistan, and Bangladesh. This sub-branch also encompasses all four sequenced Central Asian individuals of haplogroup L1-M22 in our study, as well as the most from a recent study about Central Asia.^79^ Their lineages are scattered in South Asian ones, indicating a probable origin. Genotyping analyses found that L1a2-M357/L1307 more frequently occurs among South Asian populations than in West Asian ones (Figure 1 and Table S1). Within South Asia, it is more frequent among populations from Pakistan and northern and northwestern India than in southern ones. The L1a2-Y215410 is a minor recent (∼1.3 kya) sub-branch consisting of an Armenian, a Kurdish, and an individual from Turkey. In addition, the YFull and FTDNA include one or two individuals each from Iraq, Syria, and Kazakhstan.

It is crucial to highlight that the L1a1-M27/M76 and L1a2-M357/L1307 branches exhibit strikingly similar temporal and spatial patterns. Both display sub-branches in both West Asia and South Asia, and notably, they share a coalescence time frame in the Early Holocene (Figures 2 and S1). This significant observation suggests a shared historical context or demographic events that impacted populations across these regions during this critical period.

The L1b-M317/PH982 branch presents a notable contrast (Figures 2 and S1). It is primarily distributed in West Asia (27 out of 34), more in its northern latitudes (23 out of 34). Coalescing at ∼14.2 kya, this is the oldest region-specific branch of the L1-M22. Non-West Asian members in this branch are two Makranis from Pakistan, two individuals with unknown ancestry, a Tatar from Russia, one from Poland, and one from Greece. Notably, no one from 32 L1-M22 individuals from India and Sri Lanka belongs to this branch. Genotyping analyses largely corroborate the northern West Asian concentration of this branch, adding Iranian populations to delineate the core distribution area (Figure 1 and Table S1). With few populations having L1b-M317/PH982 individuals, Pakistan, northwestern India, and Central Asia represent the periphery of the distribution. The distinct concentration of this early branch in West Asia and its age add credence to the hypothesis of L1-M22’s potential origin in this region.

**Figure 3:**
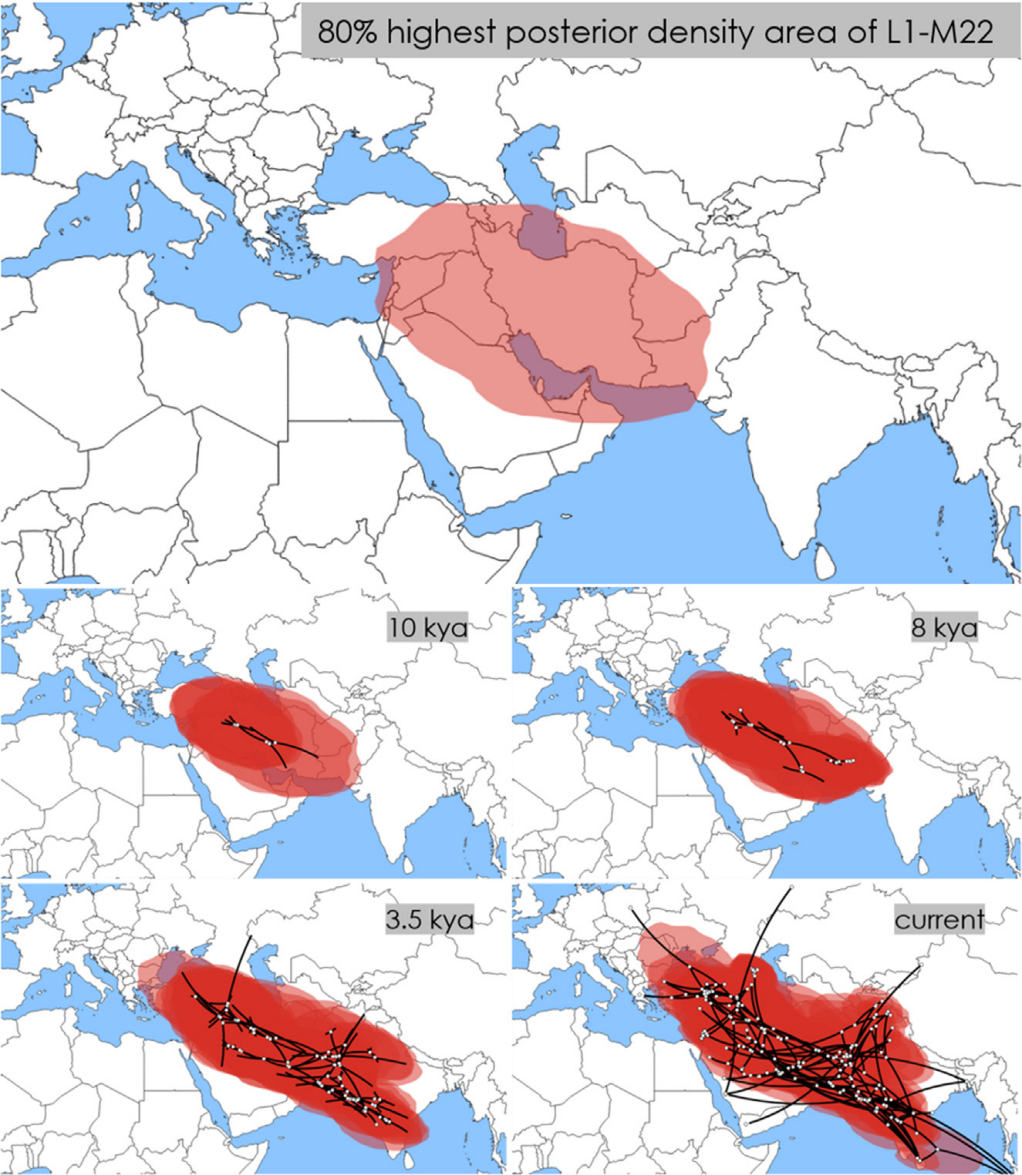
Inferred locations of the haplogroup L1-M22’s root and nodes in different time points Shaded in pink are the 80% HPD areas of the node locations inferred by Bayesian continuous phylogeographic analysis in Beast v1.10.4 software.^80^ Open circles show median estimates, while black lines indicate the branches of the maximum clade credibility tree. Maps were generated in spreaD3 software (v0.9.7.1rc).^81^ The base map was downloaded from https://github.com/johan/world.geo.json/blob/master/countries.geo.json.

We conducted a robust statistical framework to infer the place of origin of the haplogroup L1-M22 and its spread explicitly using Bayesian continuous phylogeographic analysis (Figure 3). The credible (80% HPD) area of the L1-M22 locations includes southeastern Anatolia, the Armenian Highland, the South Caucasus, the Iranian Plateau, Mesopotamia, the Levant, northern and eastern Arabian Peninsula, southwestern Pakistan, western Afghanistan, and southern Turkmenistan. At ∼10 kya, the overall area extends west toward central Anatolia and Cyprus, north to the North Caucasus, and slightly east, more in Afghanistan and Pakistan, while also covering a tiny pinch of western Gujarat in India. Importantly, more intensive diversification was occurring in and around the Fertile Crescent. At ∼8 kya, the extension continues toward Anatolia, covering almost all of it, and toward India, covering slightly more in the western region. While the earlier diversification was still ongoing in and around the Fertile Crescent, at this time, two new ones appeared in the junction area of West and South Asia. These events correspond to the L1a1-M27/M76 and L1a2-M357/L1307 branches. Both branches direct two opposing vectors to West and East. At ∼3.5 kya, the overall haplogroup L1-M22 area extends to northern, western, central, and southern India and covers Pakistan and Afghanistan almost wholly. In the west, it extends to southeastern Europe. In the south, it diffuses more in the Arabian Peninsula and covers the northeastern Sinai Peninsula. In other regions, the diffusion extended after ∼3.5 kya. To summarize, our Bayesian continuous phylogeographic analysis provides essential insights into the origin of the haplogroup L1-M22 and its diversification, which initially occurred in West Asia and then in South Asia.

**Figure 4:**
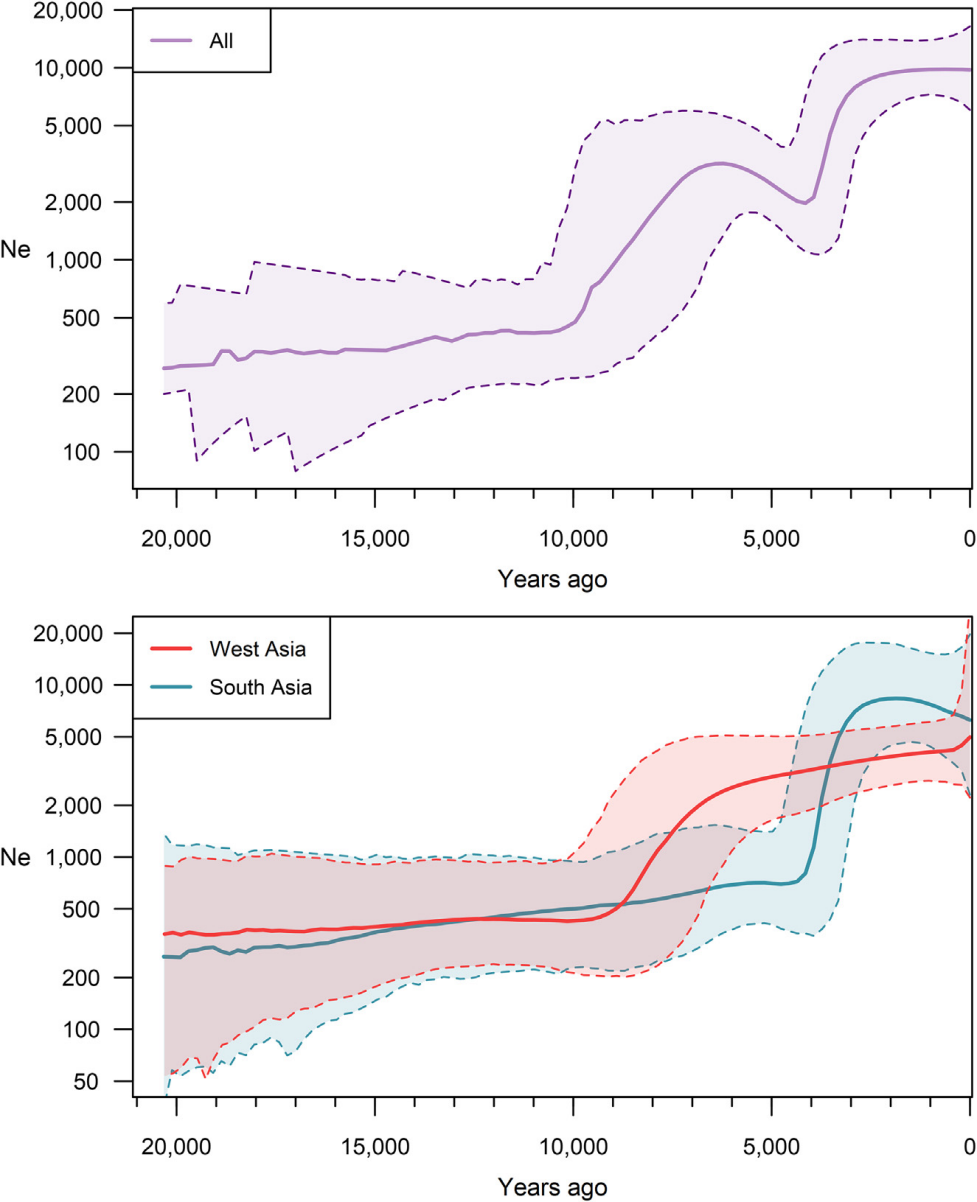
Effective population size dynamics of the haplogroup L1-M22 The solid line is the median estimate, while the dashed lines show the 95% HPD limits. Ne - effective population size.

We performed Bayesian skyline analysis to examine the dynamics of the haplogroup L1-M22’s Ne (Figure 4, upper plot). The analysis revealed that the population remained constant from the beginning until ∼10 kya. Between roughly 10 and 7 kya, Ne experienced a significant increase, indicating a potential correlation with the demographic shift during the Neolithic period. Despite a bottleneck signal from ∼7 to ∼4 kya, we consider it non-significant due to overlapping HPD intervals. From ∼4 kya onwards, the population underwent a remarkable expansion within only ∼1 ky. The lowest HPD bound at ∼3 kya surpasses the highest HPD bound of all prior epochs. Lastly, Ne shows no change over the past ∼3 kya, which can also result from the limited sample size.

To investigate whether changes in population dynamics characterize one population or are shared by both, we ran separate Bayesian skyline analyses using genetic data of individuals from only West Asia or South Asia (Figure 4, lower plot). Our results show distinct population trajectories for the two regions, with marked differences emerging ∼10 kya. In West Asia, Ne of the haplogroup L1-M22 increases starting around this time and continuing until ∼7 kya. This pattern reproduces the pattern seen during this time in the whole L1-M22 haplogroup’s Bayesian skyline analysis. It is also consistent with the intensive diversification in and around the Fertile Crescent revealed by the Bayesian continuous phylogeographic analysis described above. After ∼7 kya, Ne continues to increase, but only slightly as the HPD bounds overlap before and after this epoch.

In contrast, Ne of South Asian L1-M22 shows only a slight fluctuation between ∼10 and ∼7 kya and remains relatively stable from the beginning until ∼4 kya. Interestingly, at ∼4 kya, Ne of South Asian L1-M22 undergoes a rapid expansion within only ∼1 ky, a trend also observed in the entire haplogroup analysis and Bayesian phylogeographic analysis. Earlier research^4^ also detected an expansion of haplogroup L1-M22 in South Asia ∼4.4 kya, which coincided with that of haplogroup R1a-Z93. However, they consider this expansion weak, a conclusion likely owing to the small sample size of their study.

Ancient DNA studies extend our knowledge about the human past. However, we want to draw attention to two critical limitations that, together with low DNA preservation, affect the utility of aDNA for the haplogroup L-M20’s research. Firstly, the earliest known ancient individuals who belong to this haplogroup lived ∼6.6^74^ and ∼6.1^27^ kya (Figure S3), a time frame much later than the haplogroup’s age (>20.6 kya). Consequently, while aDNA samples offer valuable insights into more recent periods, they have limited power to inform about this haplogroup’s origin and early diversification. Secondly, our analysis of the ancient genomic data is constrained to the available haplogroup L-M20 SNPs in the “1240k capture” technology, as all the ancient genomes (Table S5), except for one, were genotyped using this approach.

The earliest so far found ancient (∼6.6 kya) individual of haplogroup L1-M22 lived in present-day Turkmenistan, close to the present-day border with Iran^74^ (Figure S3). He belongs to the L1a2-M357 branch and shares most of his autosomal ancestry with CIHG-related individuals while sharing no ancestry with AN individuals. Almost the same-age (∼6.1 kya) individuals lived in the Areni-1 cave in the South Caucasus.^27^ Interestingly, two of three individuals belong to the L1a1-Y31961 branch (Figure S2), the West Asian sub-branch of L1a1-M27/M76 in our phylogeny (Figures 2 and S1). The other individual belongs to the L1a1-M27/M76 branch and lacks reads covering SNPs that define the known downstream branches, including the L1a1-Y31961. These individuals represent a Chalcolithic population of the South Caucasus who share ancestry with CIHG (∼50%), AN (∼30%), East European hunter-gatherer (EHG) (∼10%), and LN (∼9%) populations.^51^ The next earliest (∼4.9 kya) individual of the haplogroup L1-M22 is found in the Shahr-i-Sokhta site in present-day southeastern Iran.^30^ This Bronze Age individual belongs to the L1a2-M357/L1307 (Figure S2) branch and shares all his ancestry with CIHG populations,^51^ although others in the cluster share on average ∼19% of ancestry with AN, ∼4% with Andamanese hunter-gatherer (AHG), and ∼12% with West Siberian hunter-gatherer (WSHG) populations.^30^ In this population, there are some individuals who, compared to the so-called main cluster, share more ancestry with AHG populations and lack a detectable AN-related ancestry. Similar composition is also found in some individuals from Gonur, the Bactria Margiana Archaeological Complex (BMAC) site in Central Asia. These individuals from Shahr-i-Sokhta and Gonur represent the IPC formed between ∼5400 and 3700 BCE.^30^ Current South Asian populations descend from admixture between a yet unsampled population from this cline and a population from the Central Steppe Middle Bronze Age (CSMBA). Two younger (∼3.8 kya) individuals of the haplogroup L1-M22 lived in Central Asia (Uzbekistan).^30^ Both belong to the L1a2-M357/L1307 branch. One has the derived allele for a downstream L1a2-M2398 lineage-defining SNP, the lineage to which all four present-day individuals from Central Asia affiliate. The other genome lacks reads for positions defining the downstream lineages. Although postdating, these individuals resemble individuals from the majority group of the BMAC mentioned above. That is, they share ∼59% ancestry with CIHG, ∼26% with AN, ∼2% with AHG, and ∼12% with WSHG populations.^30^ The haplogroup L1-M22 is also found among 14 ancient (∼3.0–2.3 kya) individuals from present-day Swat Valley, Pakistan.^30^ They all belong to the L1a2-M357/L1307 branch. Three were further assigned to the L1a2-Y6288, L1a2-Y17951, and Balti_YPGB066 lineages. These individuals are those that originate from admixture between IPC-related and CSMBA-related populations. An ancient (∼2.8 kya) individual from Hassanlu, present-day Iran, and another one (∼2.3 kya) from present-day Armenia^51^ share derived alleles of three and two SNPs, respectively, with a Qatari individual (SRR2098209) down to the L1a2-Y6288 lineage. They look similar in autosomal variation and share ∼56% of their ancestry with CIHG, ∼24% with LN, ∼16% with AN, and ∼3% with EHG populations. Interestingly, another ancient individual (∼1.9 kya) from present-day Armenia^82^ also belongs to the L1a2-Y6288 lineage. However, he is in a distinct sub-lineage, which includes currently living individuals from Armenia and Turkey. A Hunnic individual (∼1.7 kya) from the Tian Shan region of present-day Kyrgyzstan^83^ belongs to the L1a1-Y31961 branch. Another Central Asian individual of the same age is found in present-day Kazakhstan.^84^ He belongs to the L1a2-M357/L1307 branch and shares derived alleles for five SNPs with a Telugu individual (HG03790) from India. The earliest L1-M22 individual from Europe lived in the Medieval time (∼0.85 kya) in present-day Italy.^85^ He belongs to the L1a1-Z5926 branch.

The earliest representatives (∼5.2 kya) of the L2-L595 lineage were uncovered in the Late Maykop culture from both the Northwest and Northeast Caucasus^86^ (Figure S3). Their ancestral profile resembles that of Chalcolithic populations from present-day Armenia and Iran, as well as Kura-Araxes individuals from present-day Armenia and the Northeast Caucasus.^86^ A possible Iranian Chalcolithic representative (∼4.8 kya) is found in Tepe Hissar, a Copper Age-to-Bronze Age urban settlement in the central Iranian Plateau.^30^ However, the assignment to L2-L595 is based on only 3 C-to-T and 2 A-to-G transitions, which can also result from postmortem damage. The genetic makeup of Chalcolithic populations from present-day Armenia and Iran suggests a mixture of ancestries shared with CIHG, AN, and LN populations and a minor amount of ancestry shared with EHG/WHG population,^51^^,^^86^ likely inherited alongside the AN-related ancestry.^27^ Another ancient (∼3.9 kya) member of the L2-L595 lineage is found in the Alalakh population from the Middle-Late Bronze Age northern Levant.^31^ This population combines ancestries shared with AN, CIHG, and LN populations. Another ancient (∼3.2 kya) member is found in the Iron Age southern Levant. He shares the same three ancestry components.^87^ One more ancient (∼2.5 kya) member is from present-day Turkey’s Batman region.^88^ This individual shares ancestry overwhelmingly with CIHG and LN populations. This lineage’s most recent ancient individual is found among Vikings (∼1.1 kya) from Sweden.^85^

For two ancient L-M20 individuals, owing to low data quality, it was impossible to assign them to the downstream branches (Figure S3). One (∼4.7 kya) is from the Early Bronze Age time of present-day Greece.^89^ This population displays excessive allele sharing with earlier and contemporaneous West Eurasian groups from present-day Iran and the Caucasus. Another ancient individual (∼2.4 kya) is from the Aegean of present-day Turkey.^88^

While aware of the inherent limitations in sample ages and data availability, our analysis of ancient DNA reveals a striking consistency: the sole genetic component universally shared among all individuals within haplogroup L-M20 related to CIHG. This finding underscores a strong genetic affinity between haplogroup L-M20 and these ancient populations, at least since ∼6.6 kya.

## Discussion

This study presents extensive research on human Y chromosome haplogroup L1-M22 - the major branch of the haplogroup L-M20. Our research draws from an analysis of 165 high-coverage whole Y chromosome sequences. It employs a robust statistical framework, shedding more light on this haplogroup’s origin, distribution, and diversification. Consistent with the previously suggested estimates,^4^^,^^13^^,^^68^ it coalesces ∼20.6 kya, shortly after the L-M20 (the YFull and FTDNA). Many West Asian Y chromosome and mitochondrial DNA lineages display a pattern similar to the haplogroup L-M20: an extended bottleneck before and diversification during or shortly after the LGM. Other examples are Y chromosome haplogroups G2a-P15, J1-M267, J2a-M410, J2b-M12,^3^^,^^16^ and mitochondrial DNA haplogroups U7 and J1,^9^^,^^15^ among others. The reduced genetic diversity can be attributed to the harsh glacial conditions, which may have markedly restricted the number of founding individuals. Nevertheless, the divergence began right during the LGM, which, although surprising, aligns with growing archaeological evidence regarding the habitation of northern West Asia during the same period.^90^

Our Bayesian continuous phylogeographic analysis supports the Y chromosome haplogroup L1-M22’s origin in West Asia more than in South Asia (Figure 3). The 80% HPD area overwhelmingly encompasses West Asian regions such as the Iranian Plateau, Mesopotamia, the Armenian Highland, the Caucasus, the Arabian Peninsula, and the Levant. Although some overlap of the 80% HPD area with northwestern South Asia may suggest the origin there, two additional lines of evidence add more weight to the origin of the haplogroup L1-M22 in West Asia. Firstly, the sister branch of L1-M22, L2-L595, is found exclusively in West Asia and Europe^30^^,^^31^^,^^85^^,^^86^^,^^87^^,^^88^ (the YFull, FTDNA, Table S5, and Figures S2 and S3). Second, as detailed in the previous paragraph, the distinct pattern of a long bottleneck in the haplogroup L-M20 after splitting from haplogroup T at ∼45 kya resembles what is observed in other West Asian lineages^3^^,^^4^^,^^15^^,^^16^ and differs from the earlier divergence seen in South Asian lineages like the Y chromosome haplogroup H and mitochondrial DNA haplogroup U2, as well as others native to South Asia.^3^^,^^4^^,^^5^^,^^6^^,^^7^^,^^8^

Our results indicate that the haplogroup L1-M22 population in West Asia began to expand ∼10 kya (Figure 4). This coincides with the Neolithic demographic transition, a crucial period of human history when the world’s population started transitioning from a hunter-gatherer lifestyle to a more settled way of life based on agriculture and animal husbandry.^17^ This transition occurred earliest in the Fertile Crescent region of West Asia ∼12 kya.^20^^,^^21^^,^^22^ Centers with at least three genetically distinct population groups were revealed.^23^^,^^26^^,^^27^^,^^29^ Intriguingly, our Bayesian continuous phylogeographic analysis infers that starting at ∼10 kya, L1-M22 expands, more intensively, around the northern Fertile Crescent region (Figure 3), probably inhabited by populations with the CIHG autosomal heritage. Therefore, we propose that these populations may have contributed to the dissemination of the haplogroup L1-M22 in West Asia. This conclusion finds further support in aDNA studies, which reveal that the genome-wide ancestral component universally shared among all individuals within the haplogroup L-M20 corresponds to that shared with the CIHG populations. This observation may initially seem counterintuitive given the absence of haplogroup L-M20 in Bronze Age Pontic-Caspian steppe populations, despite these groups having half of their genetic heritage originating from a West Asian population.^53^^,^^54^ Additionally, contemporary populations with a prominent steppe ancestry, like those in northeastern Europe,^53^^,^^54^ exhibit only minimal, if any, the presence of haplogroup L-M20.^91^^,^^92^ This inconsistency can be elucidated by considering that, unlike the autosomal ancestry, the predominant paternal lineages in Bronze Age Pontic-Caspian steppe populations were inherited from earlier local populations.^30^ This marked scarcity of representation also extends to other West Asian Y chromosome haplogroups.

We did not observe an expansion in South Asian L1-M22 Y chromosomes during the Neolithic time (Figure 4). This suggests that while the West Asian L1-M22 population was expanding, likely linked to the changes during the Neolithic demographic transition, the ancestral population of the present-day South Asian L1-M22 lineages was already distinct from their West Asian Neolithic fellows. Thus, at least two populations genetically connected to the CIHG have probably emerged in West Asia around the Holocene. Groups of one population then remained in or close to the center of the Neolithic demographic transition with a lack of expansion to South Asia. Groups of another one migrated to South Asia.^30^^,^^48^^,^^50^^,^^73^ These non-Neolithic CIHG-related groups probably also involve the ancestors of the South Asian L1a1-L1320 and L1a2-M2398 branches. Individuals representing these branches migrated to South Asia starting at ∼8 to ∼6 kya. Notably, no aDNA individuals from West Asia belong to these branches, further supporting their association with South Asia. Concurrently, the L1a1-Y31961 and L1a2-Y6288 branches remained in West Asia with the L1b-M317/PH982. Interestingly, ancient individuals from the L1a1-Y31961 branch lived already at the Chalcolithic time (∼6.1 kya) in present-day Armenia. It is also interesting that an individual from the Early Bronze Age (∼4.9 kya) Shahr-i-Sokhta site in present-day southeastern Iran belongs to the L1a2-M357/L1307 branch and shares 100% autosomal ancestry with CIHG-related populations. Collectively, these findings provide further evidence supporting the association of haplogroup L1-M22 with autosomal CIHG-related ancestry, potentially indicating their role in spreading the haplogroup not only in West Asia but also into South Asia. In the latter case, though, the population had not been mixed with AN ones^30^ and shows no sign of expansion. It is important to note that the precise geographic locations of the CIHG-related populations may not align with current state borders due to historical factors.

The finding of a distinct ancient group likely closely related to CIHG during the Holocene is a crucial discovery, potentially shedding light on the origins of contemporary South Asian L1-M22 lineages. This finding corroborates the previously proposed hypothesis of a unique CIHG-related population in the eastern regions of present-day Iran and bordering regions of Central Asia.^30^ The recent discovery of a ∼6.6 kya individual with an L1-M22 lineage in present-day Turkmenistan^74^ provides additional evidence for the association of this haplogroup with a CIHG-related ancestral group to that region, which served as a junction between West and South Asia. The IPC population originated due to the admixture of this kind of population and an AHG-related population at around 5400 to 3700 BCE,^30^ a time frame overlapping closely with our age estimates of South Asian L1-M22 branches. L1-M22 lineages in a subset of ancient individuals from the IPC and later individuals from the Swat Valley^30^ reinforce this hypothesis. Our results also align with the formal modeling of that study, demonstrating that the substantial shared ancestry between present-day South Asians and early Holocene populations in present-day Iran^27^ arose as a result of a genetic influx from IPC people into later South Asians rather than a substantial westward gene flow of South Asian ancestry onto the Iranian Plateau.

Such an ancient group closely related to the IPC holds profound implications for understanding the origin and dissemination of the Dravidian family of languages, the second-largest language family in South Asia. Studies suggest that the migration of agriculture and herding from West to South Asia may have led to the introduction of proto-forms of Dravidian languages with the Neolithic migration.^36^^,^^37^^,^^38^^,^^40^^,^^41^^,^^42^ However, the absence of AN ancestry in the IPC^30^ narrows down the origin of such movement to a population from West Asia lacking AN ancestry. A recent study found notable AN ancestry in a few contemporary populations of northern India.^93^ Nevertheless, this can be linked with a more recent event: the Steppe migration. Our finding about the lack of expansion of South Asian L1-M22 lineages before ∼4 kya is consistent with the absence of a large-scale Neolithic population movement from West to South Asia. Instead, it is plausible that a population bearing only CIHG-related ancestry and L1-M22 lineages moved from West to South Asia, potentially also during the Neolithic time, but without a substantial expansion.

Within South Asia, the dispersals of Dravidian languages were most probably conducted by people with ASI ancestry,^30^^,^^62^ which was likely formed after the collapse of IVC when its people migrated eastward and southward and mixed with populations carrying higher AASI ancestry.^30^ Hence, one of the two ancestral population groups, i) the IPC or ii) the ancient eastern and southern South Asian populations with higher AASI ancestry, might be the speakers of Dravidian languages.^30^ Linguistic studies show that already IVC people may have spoken a Dravidian language.^94^^,^^95^^,^^96^

The potential Elamo-Dravidian linguistic connection^60^^,^^97^ is critically important in unraveling the origins of the Dravidian language family and its possible association with the IPC. If substantiated, a population with a CIHG-related genetic heritage would be the best candidate for disseminating both Elamite and Dravidian languages. Our study supports this hypothesis by suggesting a connection between the roots of all L1-M22 lineages and CIHG-related genetic ancestry while also delineating temporal boundaries with the reconstructed Y chromosome haplogroup L1-M22 tree. Arguably, West Asian L1a lineages likely contributed to the development of the Elamite language. In contrast, South Asian L1a lineages after ∼8 kya probably migrated from the Iranian plateau, potentially contributing to the spread of Dravidian languages to South Asia. Our results do not support the suggestion that the geographical expansion started from ancient Elam, present-day southwestern Iran. Instead, both these regions could be the final areas of migration started from yet another region inhabited by a population of CIHG-related ancestry.

The expansion of Dravidian languages into southern India aligns with the population expansion that began ∼4 kya, as observed in our Bayesian analysis of South Asian lineages of the haplogroup L1-M22. The expansion coincided with the arrival of Steppe ancestry in South Asia during the Middle and Late Bronze Age.^30^^,^^50^ Notably, Steppe ancestry-rich individuals in Central Asia belong to paternal haplogroups other than L1-M22. Consequently, local South Asian populations bearing haplogroup L1-M22 underwent population expansion simultaneously with incoming Steppe individuals as Middle and Late Bronze Age Steppe-related paternal haplogroup R1a^24^^,^^30^^,^^54^ also expanded during this time in South Asia.^4^ A similar pattern is seen also with South Asian maternal lineages.^73^ Interestingly, the beginning of population expansions followed the megadrought event that transformed several complex societies of the Bronze Age, including the IVC.^98^

This study illuminates the genetic history of Y chromosome haplogroup L1-M22. Our research emphasizes West Asia’s pivotal role in this haplogroup’s emergence and its possible association with CIHG-related genome-wide ancestry. We characterized at least two distinct population groups bearing this ancestry during the Early Holocene. One was expanding in West Asia during the Neolithic demographic transition, and the other migrated without expansion to South Asia (∼8-6 kya), possibly contributing to the spread of Dravidian languages. Importantly, our findings support the connection of Dravidian languages with ancient Elamite language spoken in present-day southwestern Iran, possibly linked to migration from West to South Asia. Nevertheless, our inferences challenge earlier claims about the dispersal of Dravidian languages in connection to the expansive spread of farming and align with the recent study about the lack of AN legacy in the ancestors of South Asians. The local South Asian L1-M22 lineages expanded between ∼4 and ∼3 kya, coinciding with the introduction of the Steppe ancestry. Hence, our research offers valuable insights into the confluence of genetic and linguistic developments during this pivotal period in South Asian history. Further interdisciplinary research is needed to fully understand these intricate patterns of the human past, integrating genetics, linguistics, and archaeology to provide a more comprehensive narrative of our shared heritage.

### Limitations of the study

Several cautions and limitations must be acknowledged when interpreting our findings. The limited sample size and potential temporal and spatial gaps in genetic data may not fully capture the diversity of West and South Asian populations, impacting the depth of insights into L1-M22’s history. It is also essential to caution against overemphasizing direct associations or causal relationships between haplogroup L1-M22 and the CIHG autosomal ancestry. Additionally, caution is advised against using a single genetic lineage as the sole representative of a language group, as populations comprise multiple lineages. Furthermore, linking genetic data with cultural and historical events, like disseminating Elamite and Dravidian languages, calls for careful consideration, as the process is complex. We hope this study serves as a foundation for further research. A comprehensive understanding of genetic and cultural dynamics in West and South Asia requires future investigations using additional genetic markers and interdisciplinary approaches. Ultimately, we emphasize that our findings cannot and must not be used to promote divisive or exclusionary narratives or to undermine or criticize individuals or groups associated with different haplogroups. Genetic data are valuable tools for understanding the human past; however, they are incompatible with being misused to oversimplify or misrepresent the rich diversity of human populations.

## STAR★Methods

### Key resources table

**Table:** 

REAGENT or RESOURCE	SOURCE	IDENTIFIER
**Deposited data**		

Raw (FASTQ) and reference mapped (BAM) reads	This study	ENA: PRJEB71943
Supplemental Figures, Tables, and Files	This study	Mendeley Data: https://doi.org/10.17632/ts4vc55rzp.1
Human reference genome NCBI build 37, GRCh37, decoy version	The 1000 Genomes Project Consortium et al.^100^	https://ftp.1000genomes.ebi.ac.uk/vol1/ftp/technical/reference/phase2_reference_assembly_sequence/hs37d5.fa.gz
Raw (FASTQ) or (BAM/CRAM) reads	The 1000 Genomes Project Consortium et al.^100^	https://www.ebi.ac.uk/ena/browser/view/PRJEB31736
Raw (FASTQ) or (BAM/CRAM) reads	Bergström et al.^13^	https://www.ebi.ac.uk/ena/browser/view/PRJEB6463
Raw (FASTQ) or (BAM/CRAM) reads	Kars et al.^101^	https://www.ebi.ac.uk/ena/browser/view/PRJNA674530
Raw (FASTQ) or (BAM/CRAM) reads	Mallick et al.^11^	https://www.ebi.ac.uk/ena/browser/view/PRJEB9586
Raw (FASTQ) or (BAM/CRAM) reads	Wong et al.^102^	https://www.ncbi.nlm.nih.gov/biosample/?term=SS6003405; https://www.ncbi.nlm.nih.gov/biosample/?term=SS6003411; https://www.ncbi.nlm.nih.gov/biosample/?term=SS6003413; https://www.ncbi.nlm.nih.gov/biosample/?term=SS6003435
Raw (FASTQ) or (BAM/CRAM) reads	Yang et al.^103^	https://ngdc.cncb.ac.cn/gsa/search?searchTerm=PRJCA000457
Raw (FASTQ) or (BAM/CRAM) reads	Karmin et al.^3^	https://www.ebi.ac.uk/ena/browser/view/PRJEB8108
Raw (FASTQ) or (BAM/CRAM) reads	Rodriguez-Flores et al.^104^	https://trace.ncbi.nlm.nih.gov/Traces/?view=run_browser&acc=SRR2098261&display=download
Raw (FASTQ) or (BAM/CRAM) reads	Carmi et al.^105^	https://ega-archive.org/search/egad00001000781
Raw (FASTQ) or (BAM/CRAM) reads	Haber et al.^106^; Gilly et al.^107^	https://ega-archive.org/datasets/EGAD00001001440
Raw (FASTQ) or (BAM/CRAM) reads	Serra-Vidal et al.^108^	https://www.ebi.ac.uk/ena/browser/view/PRJEB29142
Raw (FASTQ) or (BAM/CRAM) reads	Alsmadi et al.^109^	https://trace.ncbi.nlm.nih.gov/Traces/?view=run_browser&acc=SRR1274979&display=download
Raw (FASTQ) or (BAM/CRAM) reads	Ilyas et al.^110^	https://trace.ncbi.nlm.nih.gov/Traces/?view=run_browser&acc=SRR926184&display=download
Raw (FASTQ) or (BAM/CRAM) reads	Kaja et al.^92^	The Thousand Polish Genomes: 633_28990_20
Raw (FASTQ) or (BAM/CRAM) reads	Almarri et al.^68^	https://www.ebi.ac.uk/ena/browser/view/PRJEB28504

**Software and algorithms**		

SAMtools v1.9	Li et al.^111^	http://www.htslib.org
BEDtools v2.24	Quinlan and Hall^112^	https://bedtools.readthedocs.io/en/latest
BWA-MEM v0.7.17	Li and Durbin^113^; Li^114^	http://bio-bwa.sourceforge.net
Picard-tools-2.0.1	N/A	http://broadinstitute.github.io/picard
GATK-3.5	McKenna et al.^115^	https://gatk.broadinstitute.org/hc/en-us
BCFtools v1.6	Danecek et al.^116^	https://www.htslib.org
R v4.3.3	R CoreTeam^66^	https://cran.r-project.org/bin/windows/base/
RStudio Build 402	RStudio Team^67^	
BEAST v1.10.4	Suchard et al.^80^	https://beast.community
spreaD3 v0.9.7.1rc	Bielejec et al.^81^	https://rega.kuleuven.be/cev/ecv/software/SpreaD3
RAxML v7.3.2	Stamatakis^117^	https://github.com/stamatak/standard-RAxML
BEAGLE library v3.1.2	Ayres et al.^118^	https://beast.community/beagle
Tracer v1.7	Rambaut et al.^119^	https://beast.community/tracer
LogCombiner v1.10.4	Drummond and Rambaut^120^	https://beast.community/logcombiner
TreeAnnotator v1.10.4	Drummond and Rambaut^120^	https://beast.community/treeannotator
Surfer v8	Relethford^121^	https://www.goldensoftware.com/products/surfer/
pathPhynder	Martiniano et al.^122^	https://github.com/ruidlpm/pathPhynder
Python v3.8.0	Van Rossum and Drake^123^	https://www.python.org
FigTree v1.4.4	N/A	http://tree.bio.ed.ac.uk/software/figtree/

### Resource availability

#### Lead contact

Further information and requests for resources should be directed to the lead contact, Hovhannes Sahakyan (hovhannes.sahakyan@ut.ee).

#### Materials availability

This study did not generate new unique reagents.

#### Data and code availability

The whole Y chromosome high-coverage sequence data shared by individuals (forty-five) or generated in the current study (one) are deposited in the European Nucleotide Archive at EMBL-EBI (https://www.ebi.ac.uk/ena/browser/view) under accession ENA: PRJEB71943. They are publicly available as of the date of publication. This paper also analyzes existing, publicly available data. Accession numbers for the datasets are listed in the key resources table. Following the consent form signed by the customers of Gene by Gene commercial genetic testing company, the sequencing data included in this study is used for the sole purpose of scientific inquiry. Restrictions apply to the availability of these data, so they are not publicly available. It is reported here on an aggregate level as phylogenetic trees. In addition, the original MCC tree is provided as File S1, and the original ML tree is available as File S2. These text files can be opened using FigTree software (http://tree.bio.ed.ac.uk/software/figtree/), enabling researchers to explore the phylogenetic relationships in a user-friendly manner. Raw data from Supplemental Figures, Tables, and Files were deposited on Mendeley at Mendeley Data: https://doi.org/10.17632/ts4vc55rzp.1.

This paper does not report original code.

Any additional information required to reanalyze the data reported in this paper is available from the lead contact upon request.

### Experimental model and study participant details

#### Whole high-coverage Y chromosome sequences

In this study, all subjects included were adult males. In the phylogenetic reconstructions, we included 64 high-coverage whole Y chromosome sequences that had not been reported in academic publications. They have been all sequenced with the Illumina HiSeq 2500 platform following Y chromosome capture using a proprietary capture protocol available at Gene by Gene (Family Tree DNA) using the commercially available “BigY” service (https://learn.familytreedna.com/wp-content/uploads/2014/08/BIG_Y_WhitePager.pdf). Its targeted enrichment design utilizes 67,000 capture probes for sequencing more than 10 Mbp in the non-recombining male-specific parts of the Y chromosome at > 60× coverage.

All participants were informed about the study’s purpose and provided informed consent for their data to be used in scientific inquiry. Many of these genomes were found with the help of the Yfull and FTDNA. Ten of the high-coverage whole Y chromosome genomes belonged to individuals of haplogroup L1-M22 out of a total of 2018 male donors with self-reported ancestry from various countries, including Finland, Germany, Latvia, Lithuania, Poland, the Russian Federation, Sweden, and Ukraine. Notably, the haplogroup L1-M22 individuals in this study were from the Russian Federation. Additionally, ten genomes were obtained from individuals with Jewish ancestry. These twenty sequences were provided in scientific collaboration with the commercial genetic testing company Gene by Gene, based in Houston, Texas, USA. One more sample from our laboratory collection was sequenced using the aforementioned “BigY” service. We followed the approved guidelines by the Research Ethics Committee of the University of Tartu, and all experimental protocols were approved by the Research Ethics Committee of the University of Tartu (252/M-17). Table S2 contains detailed information on the samples analyzed in this study.

#### Published whole high-coverage Y chromosomes

From the published sources, we collected 99 high-coverage Y chromosome genomes sequenced with next-generation sequencing technologies targeting over 9 Mb regions of the chromosome (Table S2).^3^^,^^11^^,^^13^^,^^68^^,^^92^^,^^100^^,^^101^^,^^102^^,^^103^^,^^104^^,^^105^^,^^106^^,^^107^^,^^108^^,^^109^^,^^110^ If fastq reads were unavailable in the public repositories, these were extracted from BAM or CRAM genome files using SAMtools v1.9^111^ and BEDtools v2.24.^112^

#### Genotyping of SNP markers

We collected blood specimens from 707 healthy unrelated adult males who belong to three distinct West Asian populations. Patrilineal ancestors of the individuals for at least two generations belong to the populations reported here. Prior to the study, informed consent was obtained from all participants. All experimental procedures were carried out following the approved guidelines by the Research Ethics Committee of the University of Tartu. All experimental protocols were approved by the Research Ethics Committee of the University of Tartu (252/M-17).

### Method details

This study defines West Asia as including the Iranian Plateau, Mesopotamia, the Armenian Highland, the Caucasus, Anatolia, the Levant, and the Arabian Peninsula. In Table S1, we listed the populations from the Caucasus and other West Asian regions separately for ease of reading. Next, we use Anatolia with its geographic definition, marking the area west of the Anatolian diagonal.

#### Reads mapping and multi-sample variants calling

These were performed on 97 genomes sequenced with Illumina technology following the previously described protocols.^124^ The reads were mapped to the GRCh37 human reference assembly, decoy version (obtained from the 1000 Genomes Project^100^) using BWA-MEM.^113^^,^^114^ Duplicate reads were removed with Picard-tools-2.0.1 (http://broadinstitute.github.io/picard), and indel realignment was performed with GATK-3.5.^115^ The multi-sample base calling was carried out using SAMtools^111^ and BCFtools v1.6.^116^ All 97 Y chromosome genomes were mapped and called, starting with the raw fastq reads using the same script. These variants were merged^16^^,^^124^ with those called from two high-coverage haplogroup L1-M22 Y chromosomes sequenced using Complete Genomics technology (Mountain View, California) (Table S2).

#### Variant filtering

The region mask we used is detailed in our earlier study.^16^ It is based on the published regions^125^ and supplemented with high-quality regions.^3^ The latter approach minimizes platform bias after datasets are merged. We have excluded the regions (i) containing two or more subsequent singletons within 50 base pairs, (ii) having discrepant SNPs between genomes of the same individuals sequenced more than once or between paternally related individuals, (iii) having recurrent SNPs in three or more branches in the phylogeny composed of samples sequenced with the same platform, (iv) with missing data in more than 10% of samples. Moreover, we have also excluded the regions between two > 10% no-call sites if they are placed nearby and lack variants. In the end, we recovered 9,514,762 bases of the male-specific region of the Y chromosome (Table S3). We did not perform imputation because we expect platform-specific differences to be negligible with this region mask. Consistent with this, we observe a low variation of mutation rates among the branches in our initial Bayesian phylogenetic analysis with an uncorrelated relaxed clock model^126^ (Coefficient of variation = 0.0355, 95% highest posterior density (HPD) = 0.00003–0.0847).

#### Tree reconstructions and coalescence analysis

The phylogeny was reconstructed using maximum-likelihood (ML) and Bayesian Markov Chain Monte Carlo (MCMC) approaches. The ML reconstruction was performed using RAxML software version 7.3.2,^117^ with the generalized time-reversible (GTR) substitution matrix^127^ and rapid bootstrapping (n=200), followed by a subsequent ML search. Eleven members from different Y chromosome haplogroups were included to ensure proper rooting of the haplogroup L1-M22’s most recent common ancestor. All identified variants were annotated based on this phylogeny using in-house scripts and subsequently curated manually. Figure S1 displays the ML tree, while Table S4 lists the polymorphic positions and their corresponding annotations.

Coalescence time estimates were determined in the tree reconstruction with the Bayesian MCMC approach implemented in BEAST v1.10.4 software.^80^ To ensure proper rooting, we included nine members from other Y chromosome haplogroups. Three parallel analyses were run with different random number seeds, each with 50 million chains. Coalescence time estimates were inferred by providing a normal prior with a mean of 45610 and a standard deviation of 2300 to the LT node, based on a previously published estimate.^3^ We used a non-informative, uniformly distributed (1.0e^-20^ – 1.0) prior for mutation rate. For the site model, we used the GTR substitution model^127^ and the Gamma site heterogeneity model^128^ with four categories. Bayesian skyline model^129^ was used as the tree model with group sizes of 5. Population dynamics were smoothed using a piecewise-linear approach.^130^ Uniform distribution bounded by 1.0 and 1.0e^15^ was given as the skyline.popSize prior. The uncorrelated relaxed log-normal clock model^126^ was initially used (data not shown). However, all subsequent analyses were run with the Strict clock model, as variation in mutation rates between branches was negligible (0.0355, 95% HPD = 0.00003–0.0847). In the analyses, we used BEAGLE library v3.1.2^118^ for accelerated, parallel likelihood evaluation.

The results were manually inspected using Tracer v1.7 software,^119^ with satisfactory convergence being achieved as evidenced by effective sample size (ESS) values exceeding 200 for all parameters. The results of the parallel chains were combined using LogCombiner software,^120^ with a burn-in of the first 10% of records discarded. The maximum clade credibility (MCC) tree was generated with TreeAnnotator,^120^ with node heights being summarized using posterior median values. Lastly, the MCC tree was visualized in RStudio software,^66^^,^^67^ including “ggtree”,^131^^,^^132^ “ape”, “treeio”,^133^ “reshape2”,^134^ “ggplot2”,^135^ and “ggstance”,^136^ in addition to basic R packages. These procedures ensured the reliability of the results and facilitated the production of a clear and reproducible visualization of the MCC tree.

#### Bayesian phylogeography

We conducted a Bayesian phylogeographic analysis in continuous space, following established methods.^137^^,^^138^ This approach was originally designed to uncover the spatial dynamics and ancestral locations of viruses in continuous space^138^^,^^139^ but has also been applied to human Y chromosome studies.^16^^,^^91^

We selected 163 out of 165 whole high-coverage Y chromosome sequences. Two excluded genomes from the Russian Federation lacked population or geographic information and did not belong to the L1a2-M357/L1307 branch composed of Nakh-Dagestanian-speaking Northeast Caucasians.

We used the BEAST v1.10.4 software^80^ and applied molecular clock, site, and tree models and priors similar to those used in our Bayesian phylogenetic analysis described above. To infer coalescence time information, we provided a normally distributed prior with a mean of 20,600 years and a standard deviation of 100 years to the root node. This mean value corresponds to the age we estimated for haplogroup L1-M22 in this study. For the diffusion model in continuous space, we used the Brownian random walk (BRW) model.^137^^,^^140^ We ran three sets of 500,000,000 chains and ensured that ESS values were well above 200. The MCC tree was generated using a -hpd2D 0.8 flag to summarize 80% HPD area for the tree nodes. We visualized the uncertainties of the MCC tree node locations using the spreaD3_v0.9.7.1rc software,^81^ with the base world map in “geojson” format downloaded from https://github.com/Stefie/geojson-world.

#### The demographic history reconstruction

This was performed using the Bayesian skyline analysis framework.^129^ We employed a similar analysis setup to the Bayesian phylogenetic reconstruction analysis, with the exception that no outgroups were included. The dynamics of effective population size (Ne) over time were estimated using Tracer v1.7 software,^119^ and we assumed five population groups. A Bayesian skyline plot was generated in RStudio software with basic R packages.^66^^,^^67^ We used an average per-generation time of 31 years for human males.^141^^,^^142^

#### Annotation

Labeling the clades of the Y chromosome tree presents a challenge due to many available whole Y chromosome sequences and their rapid generation. Consequently, clade labels tend to be lengthy and subject to frequent changes. We follow our ML phylogenetic tree to label the clades. To simplify the labeling process, we prefer using one of the defining markers’ names for clade labels instead of long alphanumeric ones. We utilized a comprehensive source of marker information from https://ybrowse.org/gbrowse2/gff, which was downloaded on 01/Dec/2022. For widely known clades, we retained the marker names previously defined in academic publications or utilized by commercial Y chromosome trees, occasionally incorporating two of these names. For other clades, we selected the shortest name among the defining markers. To assist readers, in both the main text and Figure 2, we maintain the known alphanumeric labels up to the four-symbol levels for deeper clades while also including the known marker names. For shallower clades, we preserve the four-symbol alphanumeric part of their upstream clades and add the respective marker names. Although this approach may not be ideal, we found it optimal for this study. It avoids burdening readers with long alphanumeric labels and refers to established labels of major branches while giving a reference for all clades if necessary. The annotations can be found in Table S4, which includes not only our designated labels for the clades but also references to the labels provided by the Y Chromosome Consortium^143^^,^^144^ and the International Society of Genetic Genealogy v15.73 (ISOGG) (https://isogg.org/tree/index.html) when available. Additionally, SNP rs IDs corresponding to the dbSNP v156 database (https://ftp.ncbi.nih.gov/snp/) are provided for further identification.

#### Phylogenetically informative SNP genotyping

DNA was extracted with the published “salting out” method.^145^ We genotyped M11 or M20,^64^ M317,^46^ M27,^65^ and M357^46^ SNP markers. The alleles were identified by direct Sanger sequencing or restriction fragment length polymorphism (RFLP) analysis. The results are presented in Table S1, indicating the number of individuals associated with each lineage.

#### Spatial frequency analyses

Spatial frequency analyses were conducted with the Surfer program (version 8, Golden Software, Inc., Golden, CO, USA), following the Kriging procedure.^121^ The input data are represented in Table S1. The maps were generated using the RStudio software^66^^,^^67^ with the following packages – “lattice”,^146^ “sp”,^147^^,^^148^ “raster”,^149^ “rgdal”,^150^ “rgeos”,^151^ and “classInt”^152^ in addition to the basic packages.

#### Ancient L-M20 representatives’ affiliation

We scanned published ancient DNA studies and found individuals who belong to the Y chromosome haplogroup L (Figure S3). The information on the samples is provided in Table S5. Their affiliation to the reconstructed phylogeny was performed by the software pathPhynder.^122^ With a likelihood-based workflow in R^66^ and Python3,^123^ it takes advantage of all the polymorphic sites in the target sequence and effectively evaluates the number of ancestral and derived alleles present on each branch, then reports the most likely placement of an ancient sample in the phylogeny, together with alternatives and supporting evidence. Every informative position at critical branches was also manually looked at in the genome files.

## Supplemental information

Document S1. Figures S1–S3

File S1. Original MCC tree, related to STAR Methods and Figure 2 The original MCC tree can be opened in the FigTree software (http://tree.bio.ed.ac.uk/software/figtree/). Age estimates are in years before the present.

File S2. Original maximum-likelihood tree, related to STAR Methods and Figure S1 The original maximum-likelihood tree can be opened in the FigTree software (http://tree.bio.ed.ac.uk/software/figtree/). The FigTree software enables the visualization of various informational labels associated with different branches, enhancing the interpretability of the tree and providing a detailed perspective on the evolutionary patterns present in the dataset.

Table S1. Population frequencies of haplogroup L-M20 and its branches, related Figure 1 The populations are grouped according to the geographic regions. n1 - sample size, n2 - number of L-M20s. Underscore in the frequency columns means the marker is not genotyped, while in geographic coordinate columns, underscore means the population is not used in the spatial frequency maps.

Table S2. Whole Y chromosome high-coverage sequences used in the analyses, related to Figures 2 and S1 Sample IDs include population affiliation if the original publication explicitly states this; otherwise, the country of collection is provided.

Table S3. Recovered Y chromosome regions, related to STAR Methods In the first and second columns are the 1-based numbers of the Y chromosome regions’ start and end positions according to GRCh37/hg19.

Table S4. Annotations of the branch and marker names, related to STAR Methods and Figures 2 and S1 Branch names were annotated with the shortest marker name from the defining ones. Original positions from the reference build GRCh37/hg19 were lifted over into reference build GRCh38/hg38 and T2T positions in UCSC online genome browser. (/https://genome.ucsc.edu/cgi-bin/hgLiftOver). dbSNP ID refers to the version 156. Information about the markers, YCC_haplogroup, and ISOGG_haplogroup are taken from https://ybrowse.org/gbrowse2/gff downloaded on 01/Dec/2022. Note: Private variants have been excluded from the displayed data for specific samples due to privacy constraints.

Table S5. Ancient individuals belonging to haplogroup L-M20, related to STAR Methods and Figures S2 and S3 The samples are ordered according to the mean age.
