# Supplementary material for: Human Y chromosome haplogroup L1-M22 traces Neolithic expansion in West Asia and supports the Elamite and Dravidian connection
Source: iScience. 2024 May 17;27(6):110016. doi: 10.1016/j.isci.2024.110016 (PMC11177204; doi:10.1016/j.isci.2024.110016)

iScience, Volume *27*

Supplemental information

Human Y chromosome haplogroup L1-M22 traces Neolithic expansion in West Asia and supports the Elamite and Dravidian connection

Ajai Kumar Pathak, Hovann Simonian, Ibrahim Abdel Aziz Ibrahim, Peter Hrechdakian, Doron M. Behar, Qasim Ayub, Pakhrudin Arsanov, Ene Metspalu, Levon Yepiskoposyan, Siiri Rootsi, Phillip Endicott, Richard Villems, and Hovhannes Sahakyan

Figure S1. Haplogroup L1−M22 maximum−likelihood tree, related to Figures 2 and S2 and Tables S2 and S4

Names of defining markers are indicated on the edges of the tree. In cases where multiple markers define a branch, we have chosen the shortest one. Numbers represent the total count of markers defining each branch. Refer to Table S4 for detailed information about the markers and their associated data.

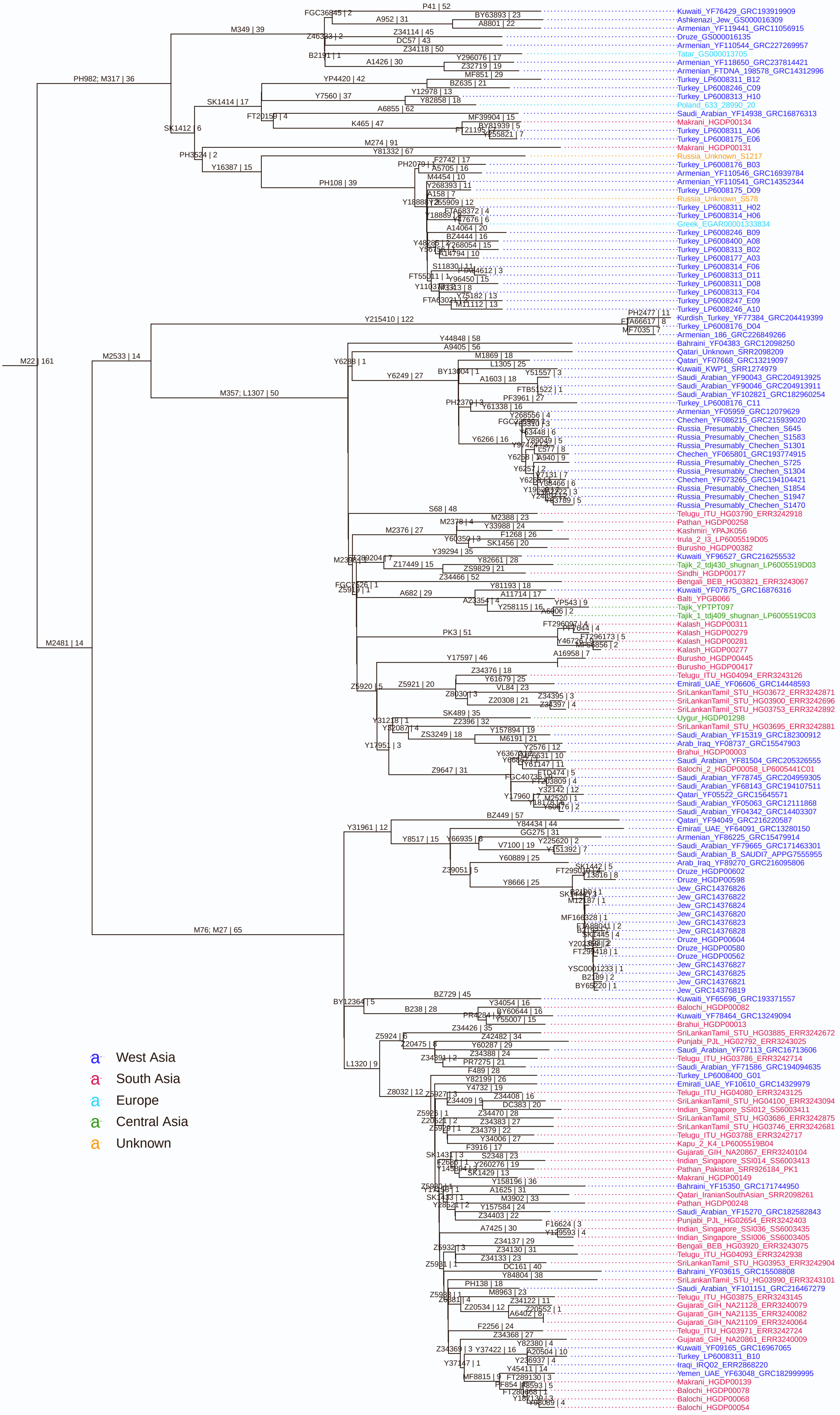

Figure S2. MCC tree with collapsed branches and ancient samples, related to Figures 2, S1, and S3 and Table S5

The MCC tree depicts the evolutionary relationships among Y chromosome sequences, with specific branches collapsed to enhance clarity. Ancient samples are denoted by diamond symbols and are positioned on the tree according to their estimated ages. Dashed lines connect each ancient sample to the specific branch it is inferred to affiliate with, as determined by the PathPhynder software. To improve visibility and prevent overlap, jitter has been applied to the diamond symbols, introducing a subtle random displacement along the vertical axis.

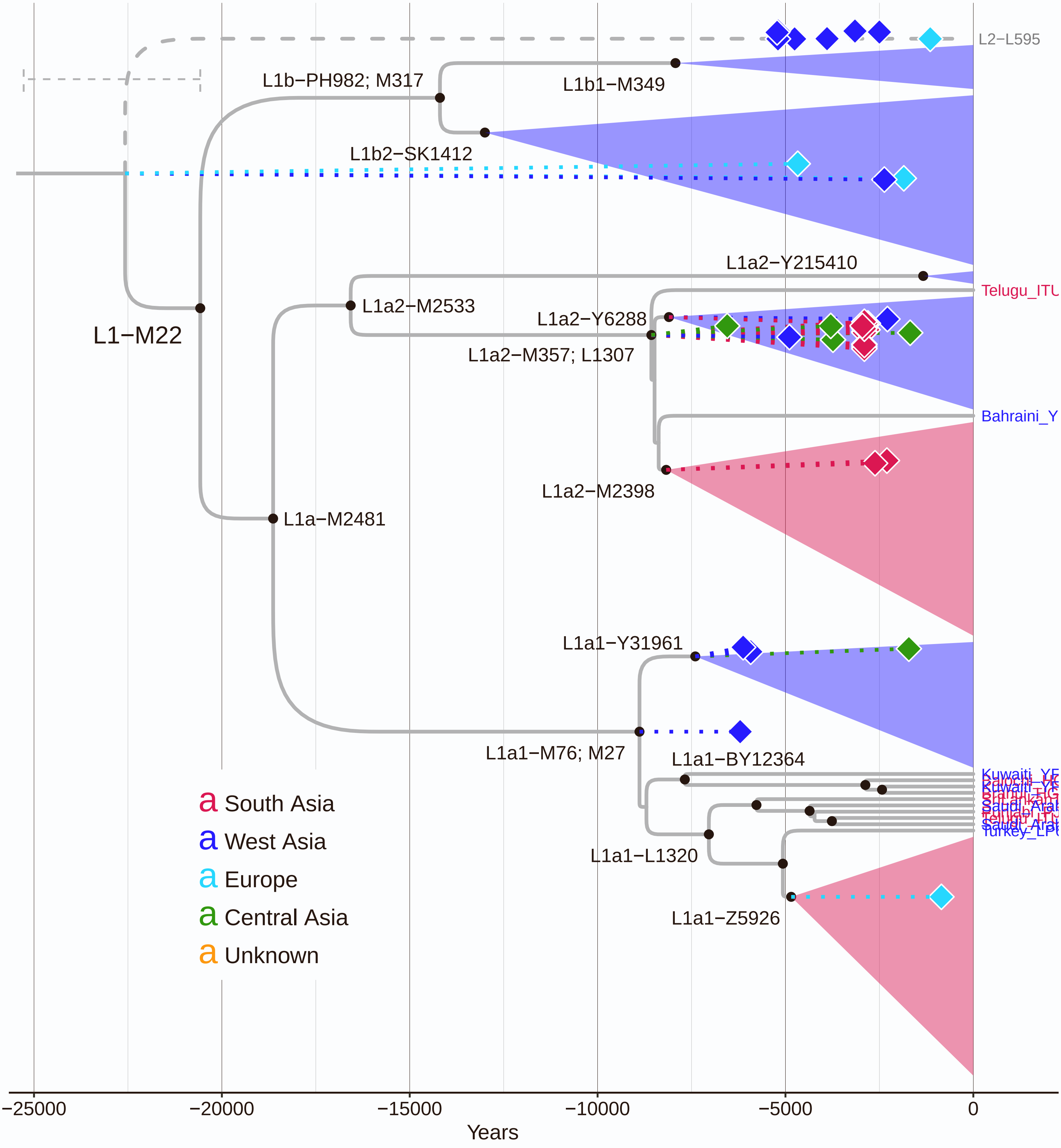

Figure S3. Ancient individuals belonging to haplogroup L−M20, related to STAR Methods, Figure S2, and Table S5

Colors mark antiquity. Detailed information about the ancient individuals is provided in Table S5. The contour map was obtained from http://tapiquen−sig.jimdo.com.

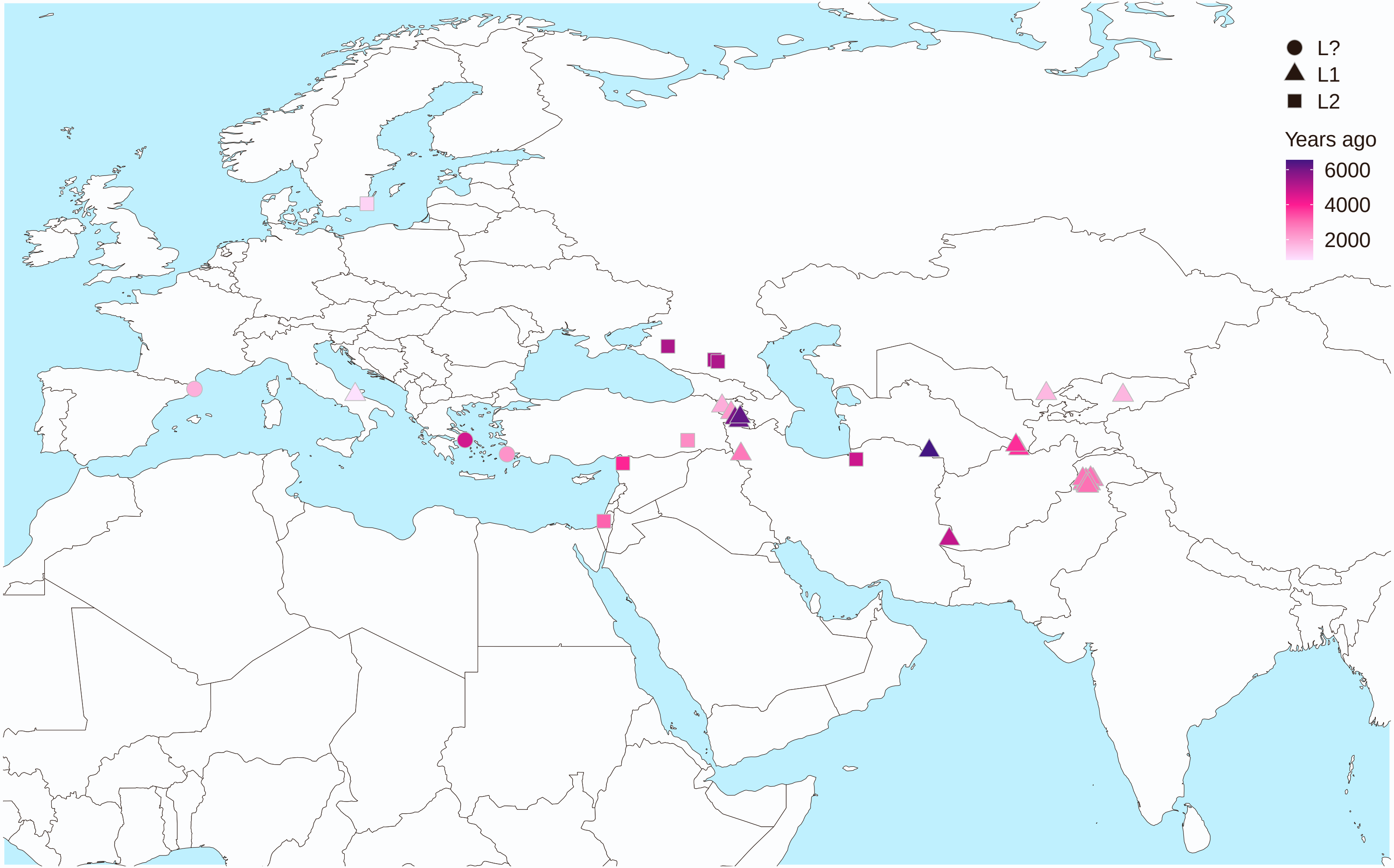

Supplement: Document S1. Figures S1–S3 [file mmc1.pdf]
